# Cerebral Toxoplasmosis, CMV and Bacterial Pneumonia with Decreasing CD4+ T-Cell Count as Results of Antiretroviral Therapy Discontinuation—A Case Report

**DOI:** 10.3390/pathogens10040497

**Published:** 2021-04-20

**Authors:** Marta Piwowarek, Katarzyna Siennicka, Tomasz Mikuła, Alicja Wiercińska-Drapało

**Affiliations:** 1 Students’ Science Society of the Department of Infectious and Tropical Diseases and Hepatology, Medical University of Warsaw, Wolska 37 Street, 01-201 Warsaw, Poland; katarzyna.siennicka1@gmail.com; 2 The Department of Infectious and Tropical Diseases and Hepatology, Medical University of Warsaw, Wolska 37 Street, 01-201 Warsaw, Poland; tomasz.mikula@wum.edu.pl (T.M.); alicja.wiercinska-drapalo@wum.edu.pl (A.W.-D.)

**Keywords:** HIV-infection, toxoplasmosis, therapy discontinuation, CD4+ T-cells, immunologic reconstruction

## Abstract

Cerebral toxoplasmosis occurs mainly in immunocompromised hosts as a reactivation of latent *Toxoplasma gondii* infection. In the diagnostic process, magnetic resonance imaging (MRI), serum testing, and biopsy are used. We describe a case of a 43-year-old HIV-positive patient presenting with altered levels of consciousness, aphasia, and hemiparesis. The patient had a history of antiretroviral therapy discontinuation for about 3 years. MRI revealed lesions, suggesting cerebral toxoplasmosis and subacute hemorrhage, serum tests for *Toxoplasma gondii* were positive. Antiparasitics and glycocorticosteroids were administered. A decline in viral load and clinical improvement were observed, however CD4+ T-cell count continued to decrease. The patient’s state worsened, he developed CMV and bacterial pneumonia, which led to his death. What is crucial in the management of an HIV-infected patient is effective and continuous antiretroviral therapy. Discontinuation of the treatment may result in AIDS and lead to poor recovery of the CD4+ T-cell population, even after reimplementation of antiretroviral therapy and a decrease in viral load.

## 1. Introduction

*Toxoplasma gondii* is an obligate intracellular parasite responsible for toxoplasmosis. In healthy adults, in the first few weeks following exposure, mild, flu-like symptoms may occur [1]. The asymptomatic state which follows is referred to as a latent infection, during which the parasite can persist lifelong within some tissues [2]. In patients with weakened immunity, reactivation of toxoplasmosis is possible and can result in a generalized infection, with neurotoxoplasmosis, also known as cerebral toxoplasmosis or toxoplasmic encephalitis, being one of the main manifestations. It typically affects patients with human immunodeficiency virus (HIV) or acquired immunodeficiency syndrome (AIDS) and is the most common cause of cerebral infection in these cases [3]. Patients develop nonspecific neurological symptoms like focal deficits, headache, and fever. Therefore, in immunosuppressed patients with neurological manifestation, cerebral toxoplasmosis should be taken into consideration in the differential diagnosis.

Reactivation of *Toxoplasma gondii* latent infection occurs mainly in immunocompromised hosts and untreated has a fatal course, as the most frequent manifestation are lesions in the central nervous system (CNS) [1,3,4]. In HIV-infected patients, reactivation typically takes place when CD4+ T-cell count decreases below 100 cells/µL, making cerebral toxoplasmosis an AIDS-defining illness [5]. However, 10% to 25% of cases have a CD4+ T-cell count above that cut-off limit [3]. It is important to note that the main cause for a sudden decrease in CD4+ T-cell count is the discontinuation of antiretroviral therapy. It occurs due to various reasons such as adverse effects of the medications or forgetfulness. However, not without significance are personal, sociological, and psychological reasons, including HIV-related stigma, that push a patient to interrupt the treatment [6,7]. 

The diagnosis of cerebral toxoplasmosis may be at times challenging. Laboratory tests such as detection of the parasite’s DNA in cerebrospinal fluid (CSF) using polymerase chain reaction (PCR) may be helpful. However, due to its low sensitivity (50–60%) [8], negative outcomes cannot exclude the diagnosis. Loop-mediated isothermal amplification (LAMP), modified PCR method, has similar sensitivity and lower sample quality requirements, but high false positive rate [9,10]. The serology does not confirm it either and may even be misleading, as the parasite seroprevalence in Europe ranges from 50% to 80%, resulting in a high number of positive IgG results in HIV/AIDS people [4,11]. Thus, differentiating between latent or reactivated forms of infection may only be possible if a significantly high IgG antibody level is found or avidity is assessed [11]. When it comes to IgM antibodies, they are usually absent during the reactivation of toxoplasmosis [12]. Moreover, some authors point out that negative test results do not exclude infection as up to 13% of AIDS-related CNS toxoplasmosis cases come out seronegative [3].

Magnetic resonance imaging (MRI) is commonly used in the diagnosis of toxoplasmosis, as some particular MR images are highly characteristic of this disease [13]. Most frequent findings include multiple hypodense rings or nodular-enhancing lesions with perilesional edema [3,4]. However, these lesions are of low specificity, since comparable alterations may be found in pathologies of different etiology [3]. Certain typical localizations of the lesions such as basal ganglia and frontal lobe may be suggestive of the diagnosis. Nonetheless, a broad spectrum of CNS toxoplasmic lesions localization has been reported, including the cerebellum and spinal cord. Moreover, neurotoxoplasmosis is the most frequent cause of supratentorial or infratentorial single lesions in HIV patients [3,14].

## 2. Case Report

A 43-year-old Caucasian man was admitted to the neurology ward due to altered levels of consciousness, aphasia, and hemiparesis. Owing to the history of HIV infection and focal changes in computed tomography and MRI, he was transferred to our Department of Infectious and Tropical Diseases and Hepatology three days later.

The patient had been infected with HIV for about 15 years and had been treated with combined antiretroviral therapy (cART) consisting of tenofovir, lamivudine, saquinavir, and ritonavir. Importantly, he had a history of therapy discontinuation. In 2013, his HIV viral load had been undetectable with a CD4+ T-cell count of 607 cells/µL (Table 1). The therapy cessation had occurred about 3 years later, that is in 2016. The patient had resigned from treatment for personal reasons, as he believed it would have affected his relationships and made him appear weaker in the eyes of the loved ones. A follow-up examination had found a viral load of 29,342 copies/mL and a CD4+ T-cell count of 329 cells/µL, yet the patient had not resumed his therapy.

**Figure 1:**
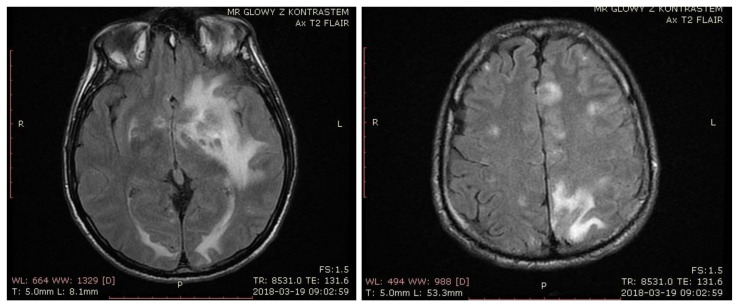
T2-weighted brain MRI from the day of admission presenting multiple hyperintense lesions and perilesional edema with mass effect.

In March 2018, the patient presented with altered levels of consciousness, aphasia, and hemiparesis. On admission to the neurology ward, he underwent MRI, which on T2 and fluid-attenuated inversion recovery (FLAIR), revealed multiple hyperintense lesions and perilesional edema with mass effect, located in both cerebral hemispheres and subcortically (Figure 1). The largest lesions were situated in deep brain structures and white matter of the left frontal, temporal, and parietal lobes. T1-weighted imaging showed a hyperintense lesion located in basal ganglia, suggesting an acute hemorrhagic transformation of ischemic changes with probable subacute hemorrhage. A peripheral post-contrast enhancement of the lesions was also present. Additionally, solid–liquid lesions were found in the left hemisphere. Diffusion-weighted imaging (DWI) found regions of restricted diffusion in deep brain structures in the area of the caudate nucleus, left frontal horn, left temporal lobe, and both parietal lobes. 

Three days later, the patient was transferred to our department in a serious condition, presenting with neurological symptoms such as confusion, aphasia, central facial palsy, right-sided hemiparesis, mild paresis in the upper left extremity, and inability to walk. A blood cell count was performed, showing 28 cells/µL for CD4+ and 444 cells/µL for CD8+ T-cells, with CD4+/CD8+ ratio of 0.06. HIV RNA testing revealed a viral load of 400,313 copies/mL. Antiretroviral therapy was initiated with emtricitabine, tenofovir, darunavir, and ritonavir. However, due to detected tenofovir resistance, treatment was changed to abacavir, lamivudine, darunavir, and ritonavir. The presence of the gene HLA-B*5701 was excluded. 

Cerebrospinal fluid was obtained, and microbial diagnostics was performed excluding cerebral tuberculosis, neurosyphilis, and *Cryptococcus neoformans* infection. Apart from an elevated protein level (1.40 g/L) and positive Nonne–Apelt and Pandy’s reactions, the analysis of CSF revealed no abnormalities (cytosis 2 cells/µL and glucose 4.38 mmol/L). Serum testing for anti-HCV (hepatitis C virus) antibodies, as well as HBs (hepatitis B surface) antigen and anti-HBc (hepatitis B core) total antibodies, was negative, whereas the serum IgG antibodies for *Toxoplasma gondii* came out positive, confirming the diagnosis of cerebral toxoplasmosis. Another MRI was performed, confirming previous findings. An ophthalmologist consultation found no abnormalities of the eyeground.

A stereotactic biopsy was considered, however it could not have been conducted due to hemorrhagic changes and high operative risk as assessed by a consulting neurosurgeon. Based on positive serology and the MRI features indicative of neurotoxoplasmosis, as interpreted by a group of radiology experts, toxoplasmosis was diagnosed. Treatment with pyrimethamine, sulfadiazine, and folinic acid as well as administration of glycocorticosteroids was initiated. After a few days of the therapy, the patient’s neurological status improved, and he began physiotherapy involving walking. Despite that, speech impairment, central facial palsy, and mildly impaired motor functions persisted. 

**Figure 2:**
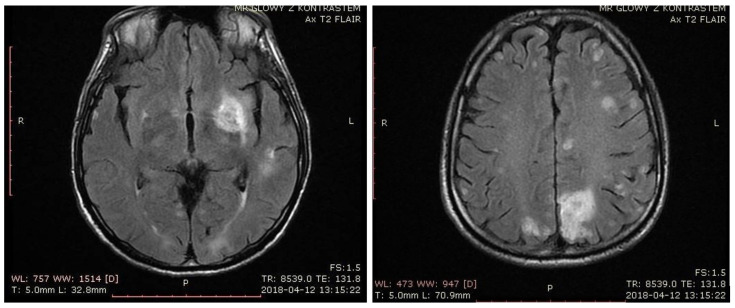
MRI performed after three weeks of treatment showing reduced perilesional edema and sharper borders in some of the lesions.

The third week of toxoplasmosis treatment brought aggravation of the patient’s state. He could no longer walk, he subsequently presented with dysphagia, bulbar palsy, tetraparesis, positive Babinski sign on the left side, and his condition was described as serious. The third MRI was performed. As compared to the previous ones it revealed significantly reduced perilesional edema and sharper borders in some of the lesions (Figure 2). That sign of improvement might have indicated toxoplasmosis as a more probable etiology, however other lesions remained unchanged, including T1 finding, suggesting subacute hemorrhage. 

After four weeks of antiretroviral treatment, the patient’s viral load was 264 copies/mL. Still, a progressive decrease in CD4+ T-cell count from 28 to 4 cells/µL was being observed. As his general condition worsened, IL-6, C-reactive protein, and procalcitonin levels increased indicating ongoing infection.

The patient underwent a chest X-ray which exposed diffuse bilateral interstitial infiltrates with partially overlapping parenchymal changes in the right upper lobe. Even though radiological findings suggested pneumocystis pneumonia, bronchoalveolar lavage culture confirmed *Acinetobacter baumani* and *Enterobacter cloacae* producing extended-spectrum beta-lactamases (ESBL), susceptible only to imipenem (*Enterobacter cloacae*) and colistin (both bacteria), whereas real-time PCR detected cytomegalovirus (CMV) RNA in serum. Following antibiogram intravenous and inhaled colistin, imipenem/cilastatin, as well as acyclovir and ganciclovir, were administered. 

Despite the implemented therapy the infection did not subside. Although subsequent chest X-rays showed regression of the parenchymal changes within the right upper lobe, new ones appeared in both lower lung fields and left middle field and interstitial abnormalities remained unchanged. The patient’s state critically worsened, eventually leading to respiratory insufficiency and death. An autopsy was not conducted, honoring the patient’s family’s will to refrain from it. 

## 3. Discussion

Although toxoplasmosis can be characterized by some highly suggestive features, there is considerable overlap with primary CNS lymphoma (PCNSL). These are the two most frequent causes of brain lesions with perilesional edema and mass effect, making the diagnostic process more complex [15]. Clinical presentation cannot differentiate between any of these diseases since a variety of neurological symptoms, including these of our patient, may occur in both. Certain MRI findings such as ring-enhancing lesions are also highly similar, although they are observed with lesser frequency in lymphoma. Their typical localization includes deep brain structures and basal ganglia for both diseases. PCNSL alterations in MRI usually tend to have better-defined borders as compared to those found in toxoplasmosis and rarely localize in the posterior fossa. This corresponds to changes in the described case, indicating that the diagnosis of PCNSL is less likely. Nevertheless, none of the mentioned characteristics can discriminate those conditions specifically [16].

In debatable cases, a stereotactic biopsy ought to be considered [5]. Nonetheless, the patient’s state needs to be stable in order to conduct such an invasive procedure, and therefore, our patient could not have been qualified for it. Additionally, administration of corticosteroids may lead to false-negative biopsy results in lymphoma cases, thus in the case when toxoplasmosis cannot be excluded, early implementation of antiparasitic treatment seems to be reasonable management [16].

What draws attention in our patient’s case is a progressive decrease in CD4+ T-cell count despite effective antiretroviral therapy resulting in a decline of viral load. The cause of such a course of the disease is unknown. Some authors indicate an immunosuppressive effect of CMV reactivation and its impact on mortality in HIV-patients with poor CD4+ T-cell recovery [17,18]. It has been suggested that CMV may increase the vulnerability of CD4+ T-cell by cellular activation, which makes them more susceptible to HIV and by limiting the antiviral effector functions of CD8+ T-cell [19]. On the other hand, delayed reconstruction of CD4+ T-cell compartment has been observed in the following groups: elderly, patients with lower baseline CD4 T-cell counts, and among those with discontinuous antiretroviral therapy [20]. Additionally, it has been proposed that impaired thymic output and naive CD4+ T-cell depletion may contribute to immune failure, but what plays an even more important role, is CD4+ T-cell nadir, especially in those patients with CD4 T-cell count <200/µL [21]. In our patient’s case, those theories may partially explain the low CD4+ T-cell count. However, presumably, other unknown factors might have played a part in the lack of immunologic reconstruction. 

Nevertheless, regardless of the reason for immune failure, patients whose CD4+ T-cell population does not rebuild despite antiretroviral treatment are at higher risk of death [21]. What also plays a significant role in higher morbidity and mortality in HIV-patients, is the discontinuation of treatment, especially in those with low CD4+ T-cell count, high viral load, or history of AIDS [22]. The benefits of the treatment in the form of viral load reduction, a sufficient increase of CD4+ T-cell count, and immunologic reconstruction do not last longer than 24 months. Moreover, the risk of AIDS/death doubles within 3 months of discontinuation [22,23]. In our patient’s case, the cessation of the therapy was a direct cause of a decrease in CD4+ T-cell count and an increase of viral load, which lead to numerous opportunistic infections, and potentially, to the lack of immunological reconstitution. Given all that, a successful antiretroviral therapy shall not be discontinued without an apparent medical reason [24].

In the described case, the diagnostic process was hindered by its limitations including contradictions to the biopsy, unclear MRI findings, and difficulty in assessing the cause of the clinical presentation. An autopsy might have provided needed answers that could be useful both for us and the scientific community. However, since it could not be performed, what can be learned from this case is that the management of AIDS patients may take an unexpected turn and despite the efforts of the caregivers, certain aspects of the patients’ disease remain unknown. Our case proves that factors influencing the immunological reconstruction and CD4+ T-cells population recovery are not yet sufficiently understood and need further investigation.

## 4. Conclusions

During antiretroviral management of an HIV-infected patient, it is essential that successful treatment must not be discontinued, as it may result in AIDS or opportunistic diseases and even lead to death. Therefore, caregivers must provide adequate information to the patient and emphasize the risks arising from therapy cessation.

## Figures and Tables

**Table 1:** Summary of CD4+ T-cells count and viral load changes over time.

Date	CD4+ T-Cells [Cells/µL]	Viral Load [Copies/mL]
July 2013	607	undetactable
May 2016 (short, unknown time after therapy cessation)	329	29,342
March 2018 (on admission)	28	400,313
April 2018 (after 4 weeks of treatment)	4	264

## Data Availability

Not applicable.
